# Incidence and Cost of Acute Kidney Injury in Hospitalized Patients with Infective Endocarditis

**DOI:** 10.3390/jcm8070927

**Published:** 2019-06-27

**Authors:** Victor Ortiz-Soriano, Katherine Donaldson, Gaixin Du, Ye Li, Joshua Lambert, Mark Rudy, Dan Cleland, Alice Thornton, Laura C. Fanucchi, Moises A. Huaman, Javier A. Neyra

**Affiliations:** 1 Division of Nephrology, Bone and Mineral Metabolism, Department of Internal Medicine, University of Kentucky Medical Center, Lexington, KY 40506, USA; 2 Center for Health Services Research, University of Kentucky, Lexington, KY 40506, USA; 3 Department of Statistics, University of Kentucky, Lexington, KY 40506, USA; 4 Division of Infectious Diseases, Department of Internal Medicine, University of Kentucky Medical Center, Lexington, KY 40506, USA; 5 Division of Infectious Diseases, Department of Internal Medicine, University of Cincinnati, Cincinnati, OH 45221, USA

**Keywords:** acute kidney injury, infective endocarditis, healthcare costs, opioid use

## Abstract

Acute kidney injury (AKI) is a frequent complication of hospitalized patients with infective endocarditis (IE). Further, AKI in the setting of IE is associated with high morbidity and mortality. We aimed to examine the incidence, clinical parameters, and hospital costs associated with AKI in hospitalized patients with IE in an endemic area with an increasing prevalence of opioid use. This retrospective cohort study included 269 patients admitted to a major referral center in Kentucky with a primary diagnosis of IE from January 2013 to December 2015. Of these, 178 (66.2%) patients had AKI by Kidney Disease Improving Global Outcomes (KDIGO) serum creatinine criteria: 74 (41.6%) had AKI stage 1 and 104 (58.4%) had AKI stage ≥2. In multivariable analysis, higher comorbidity scores and the need for diuretics were independently associated with AKI, while the involvement of the tricuspid valve and the need for vasopressor/inotrope support were independently associated with severe AKI (stage ≥2). The median total direct cost of hospitalization was progressively higher according to each stage of AKI ($17,069 for no AKI; $37,111 for AKI stage 1; and $61,357 for AKI stage ≥2; *p* < 0.001). In conclusion, two-thirds of patients admitted to the hospital due to IE had incident AKI. The occurrence of AKI significantly increased healthcare costs. The higher level of comorbidity, the affection of the tricuspid valve, and the need for diuretics and/or vasoactive drugs were associated with severe AKI in this susceptible population.

## 1. Introduction

Infective endocarditis (IE) is a severe infectious process that carries high morbidity and mortality [1,2,3]. Reports indicate that the incidence of IE in the United States has been increasing steadily in recent years [4,5]. The crude incidence has increased from 7.6 to 9.3 cases per 100,000 persons annually from 1998–2013 [5]. Moreover, healthcare costs for patients with IE increased eighteen-fold, from $1.1 million in 2010 to $22.2 million in 2015 [6].

In the United States, some areas such as the Midwest and West have exhibited a steady increase in the incidence of IE in relation to the growing incidence of opioid dependence [7]. According to the National Survey on Drug Use and Health, persons with opioid and other substance use disorder who inject drugs are at higher risk of blood-borne infections and IE [8]. Infectious complications are common and constitute a major cause of hospitalizations [9].

Acute kidney injury (AKI) is a syndrome that occurs in about 20% of hospitalized patients [10,11]. AKI is also associated with high morbidity and mortality and affects hospital resource utilization and healthcare costs [12,13]. Patients with IE can develop distinct forms of acute or subacute kidney disease such as glomerulonephritis, infarction or cortical necrosis [14,15], manifesting as AKI [16]. Prior studies have shown that older age and the degree of thrombocytopenia were associated with an increased risk of AKI in patients with IE [17]. Moreover, some antibiotics commonly used to treat IE (e.g., aminoglycosides, vancomycin, etc.) can exert toxicity to the kidneys under specific circumstances [18].

The main objective of this study was to examine the incidence and clinical parameters associated with AKI in hospitalized patients with IE in Kentucky, a state highly impacted by the opioid epidemic. We also examined hospital costs associated with the occurrence of AKI and major adverse kidney events (MAKE) in this susceptible population.

## 2. Materials and Methods

### 2.1. Study Design and Participants

Single-center, retrospective cohort study. The study population included adult patients 18 years or older who were admitted to the University of Kentucky Albert B. Chandler Hospital with IE as the primary diagnosis. Patients were excluded if they had a history of end-stage kidney disease (ESKD), baseline estimated glomerular filtration rate (eGFR) less than 15 mL/min/1.73 m^2^, were recipients of a kidney transplant, or if they had a prior episode of IE that required hospitalization. The study period was from January 2013–December 2015. The study was approved by the University of Kentucky Institutional Review Board.

### 2.2. Study Variables and Definitions

Data were gathered through automated and manual extraction from electronic health records (EHRs) and validated through comprehensive individual review of all records. The presence of comorbidities at the time of index hospitalization was assessed using the Elixhauser score [19], with individual comorbidities identified using ICD-9/10-CM codes. IE as the primary diagnosis was defined by the modified Duke Criteria [20]. IE characteristics including the location, type and number of affected cardiac valves were obtained by individual review of EHRs (consult notes, echocardiography reports, etc.). Baseline eGFR was estimated using the Chronic Kidney Disease Epidemiology Collaboration (CKD-EPI) equation [21], and baseline serum creatinine (SCr) was assessed as follows: (1) Outpatient SCr value closest to index admission within 1 year of hospitalization; if unavailable, (2) last inpatient SCr value from prior hospitalization within 1 year of index admission; if unavailable, (3) lowest SCr value throughout the index hospitalization. We gathered data of substance use disorder specific for opioid and IV drug use with the following ICD-9-CM codes: 292.0–9, 304.00–93, 305.20–93, 648.30–34, 655.50–53, 760.72–75, 779.5, 965.00–09, and V654.2. We extracted medication exposure, procedure data, and total costs related to the index hospitalization from the hospital billing system. The University of Kentucky Albert B. Chandler Hospital utilizes Allscripts Sunrise Clinical Manager™ EHR software (Allscripts, Chicago, IL, USA). Microbiologic data were obtained from blood, valve, abscess fluid, pleural fluid and bone biopsy.

### 2.3. Study Outcomes

#### 2.3.1. Clinical Outcomes

The primary outcome was incident in-hospital AKI defined and graded by the Kidney Disease Improving Global Outcomes (KDIGO) SCr-criteria. [22] We grouped AKI stages 2 and 3 due to the low numbers in each category in relation to AKI stage 1 and no AKI. A secondary outcome was MAKE, a composite of all-cause mortality, dependence on renal replacement therapy (RRT), or inability to recover at least 50% (MAKE50) or 25% (MAKE25) of baseline eGFR (if not on RRT) up to 90 days after hospital discharge [23]. Other secondary outcomes included hospital mortality and hospital readmission due to recurrent or a new episode of IE [24,25].

#### 2.3.2. Healthcare Cost Outcomes

The healthcare cost outcome was the median total direct cost of hospitalization in dollars. Other outcomes included hospital length of stay (days) and total days in a telemetry or intensive care unit (ICU) bed.

### 2.4. Statistical Analysis

Categorical data were reported as number of observations (percentages) and continuous variables as mean ± standard deviation (SD), or median (1st quartile (IQ1)—3rd quartile (IQ3)) as appropriate. Comparisons between groups for categorical variables were made using chi-squared test or Fisher’s exact test. Continuous variables were compared using the ANOVA or Kruskal-Wallis test as appropriate.

Multivariable logistic regression modeling was used for AKI stage 1 (vs. no AKI), AKI stage ≥2 (vs. no AKI or AKI stage 1), and MAKE (50% or 25%) as dependent variables and included candidate independent variables that were statistically significant in the bivariate analysis and/or carried clinical significance for the association with the dependent variables. The model section was performed using stepwise regression with a cutoff of 0.1 for variables to enter the model and 0.05 to be removed. All statistical analyses were performed using SAS 9.3 (SAS Institute, Charlotte, NC, USA) with an alpha level set at *p* < 0.05 (two-tailed) for all comparisons.

## 3. Results

### 3.1. Clinical Characteristics

**Figure 1:**
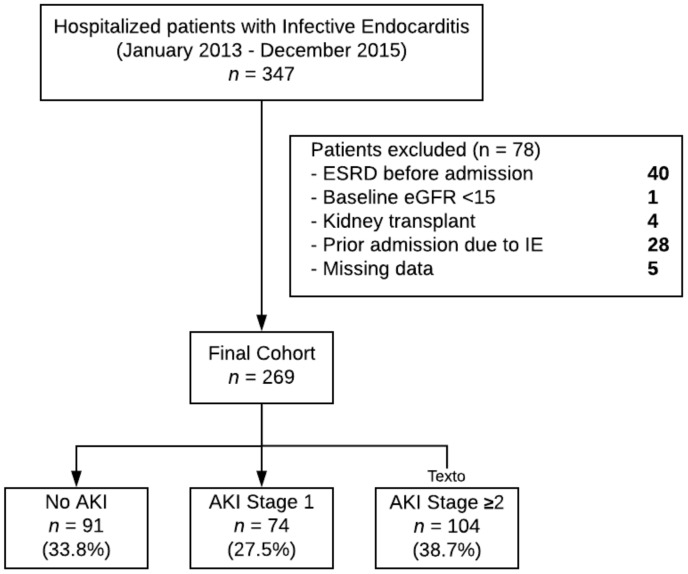
Cohort derivation. Abbreviations: AKI (acute kidney injury), eGFR (estimated glomerular filtration rate), ESRD (end-stage renal disease), IE (infective endocarditis).

A total of 269 patients were included in the study. The cohort derivation algorithm is detailed in Figure 1. The mean (SD) age was 45.4 (16.2) years, 59.5% were male, and 95.5% white. The overall incidence of AKI was 66.2%, 74 (41.6%) patients had AKI stage 1 and 104 (58.4%) patients had AKI stage ≥2. The frequency of substance use disorder was not significantly different according to AKI status (54.5% in patients with AKI vs. 50.5% in patients without AKI, *p* = 0.540). Patient’s clinical characteristics according to AKI status are detailed in Table 1. 

### 3.2. Clinical Parameters Associated with AKI

When compared with patients without AKI, patients with AKI had higher Elixhauser comorbidity scores (median (IQ1-IQ3) 4.0 (3.0–6.0) for no AKI, 6.0 (4.0–7.0) for AKI stage 1 and 7.0 (5.0–8.0) for AKI stage ≥2, *p* < 0.001), more likely to have hepatitis B and C coinfection, sepsis, and tricuspid valve involvement (21.9% for no AKI, 24.7% for AKI stage 1 and 45.2% for AKI stage ≥2, *p* < 0.001), and required more frequently diuretics and vasopressor/inotrope support. Patients with AKI more frequently had valve replacement interventions (17.6% for no AKI, 18.9% for AKI stage 1 and 31.7% for AKI stage ≥2, *p* = 0.037) (Table 1). Patients with AKI were less likely to receive angiotensin converting enzyme inhibitors (ACEI) or angiotensin receptor blocker (ARB) (39.6% for no AKI, 16.2% for AKI stage 1 and 20.2% for AKI stage ≥2, *p* < 0.001). There was no difference among the IE causative organisms between patients with and without AKI (Table 1).

In multivariable analysis, higher Elixhauser comorbidity score, more frequent exposure to diuretics and non-steroidal anti-inflammatory drugs (NSAIDs), and less frequent exposure to an ACEI/ARB were independently associated with AKI. Further, requiring vasopressor/inotrope support and having tricuspid valve involvement independently associated with AKI stage ≥2 (adjusted OR of 4.94, 95% CI: 1.52–16.01, *p* = 0.008 and 2.97, 95% CI: 1.59–5.51, *p* < 0.001, respectively) (Table 2).

### 3.3. Clinical Outcomes Associated with AKI

Patients with AKI stage ≥2 had slightly higher hospital mortality rates than those without AKI or AKI stage 1 (19.2% vs. 13.3%, *p* = 0.194). The frequency of MAKE50 was significantly higher in patients with AKI vs. those without AKI (38.5% for no AKI, 54.1% for AKI Stage 1, and 59.6% for AKI Stage ≥2, *p* = 0.011) (Table 3). Patients with AKI had also a slightly higher frequency of MAKE25 than those without AKI (71.9% vs. 60.4%, *p* = 0.125). There was no difference in hospital readmission rates due to IE according to AKI classification (Table 3).

In multivariable analysis, the occurrence of sepsis as a complication of IE was independently associated with MAKE50 (adjusted OR 2.03, 95% CI: 1.23–3.36, *p* = 0.006). Further, AKI stage ≥2 had a borderline significant association with MAKE50 (adjusted OR 1.97, 95% CI: 1.09–3.58, *p* = 0.126) (Table 4).

### 3.4. Healthcare Costs Outcomes

**Figure 2:**
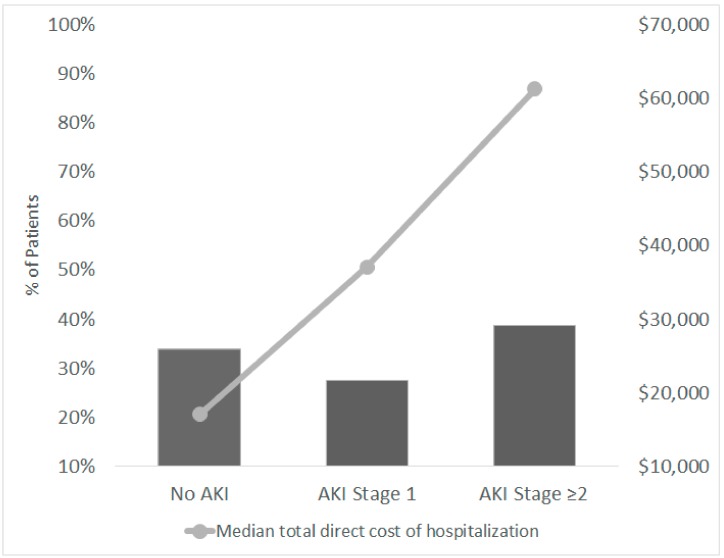
Incidence of acute kidney injury and median total hospitalization direct cost in hospitalized patients with a primary diagnosis of infective endocarditis.

The median cost of hospitalization was $35,552. The median cost of hospitalization in the AKI group was three times higher than in the no AKI group ($52,654 (25,846–73,946) vs. 17,069 (6722–31,910), *p* < 0.001) and increased accordingly to AKI severity (Figure 2 and Table 3). Patients with AKI had a longer length of hospital stay (median (IQ1–IQ3) 9.0 (5.0–17.5) days for no AKI, 23.0 (12.0–43.8) for AKI stage 1 and 34.5 (16.8–48.0) for AKI stage ≥2, *p* < 0.001). Similarly, patients with AKI had a longer length of ICU stay and more days on telemetry beds when compared with those without AKI (Table 3).

## 4. Discussion

The main finding of our study is the high incidence of AKI in a large cohort of hospitalized patients with IE. About 2 out of 3 patients (66.2%) suffered from an episode of AKI while in the hospital, which translated into higher resource utilization and healthcare costs. The median cost of hospitalization was 3.6 times higher in patients with severe AKI (stage ≥2) vs. those without AKI. We also found that tricuspid valve involvement, the need for vasopressor/inotrope support and diuretics, and a higher level of comorbidity were associated with severe AKI (stage ≥2).

Other studies have also reported the incidence of AKI in patients with IE. Ritchie et al., [18] studied a cohort of 211 patients diagnosed with bacterial endocarditis at the Brigham and Women’s Hospital between January 2009 and October 2013. They reported a lower incidence of AKI of 38.9% (only 15.2% of patients in their cohort had substance use disorder vs. 53.2% in our cohort). In contrast, our study used the KDIGO SCr-criteria to define AKI, while they used the Acute Kidney Injury Network’s (AKIN) SCr and urine output criteria [26]. Furthermore, in our cohort, patients had overall higher comorbidity scores in comparison to the referred study. In addition, Boils et al., [16] reported a biopsy-based pathologic series of 49 patients who had kidney function impairment in the setting of IE. The most common presentation of kidney disease in this population was AKI (79% of the cases) and the most common kidney biopsy findings were necrotizing and crescentic glomerulonephritis (53%) and endocapillary proliferative glomerulonephritis (37%). In addition, acute tubular injury was present in 86% of the cases.

Patients with severe AKI (stage ≥2) had slightly higher hospital mortality rates than those without AKI or AKI stage 1. Further, patients with severe AKI (stage ≥2) had significantly higher rates of major adverse kidney events than patients without AKI. Emerging evidence suggests that not only those patients with clinical evidence of AKI but also approximately 20% of patients that do not meet SCr-criteria of AKI still have increased risk of developing acute kidney disease (persistent alterations in kidney function or structure within the next 3 months following an episode of AKI), which is associated with incident or progression of CKD, ESKD, and death [27,28]. It is possible that some patients in the no AKI group may have had subclinical AKI [29] not detected by SCr changes precluding a more pronounced differentiation in clinical outcomes among those with vs. without AKI. In addition, the observed absence of mortality differentiation according to incident AKI or AKI severity stages may be due to the low event rate and lack of power. Overall rates of hospital mortality in patients with IE reported in other studies range between 18% and 24% [30,31,32], similar to the hospital mortality rate reported in our study (15.6%). Further, Wallace et al., [32] studied a cohort of 208 patients with IE and reported that mortality at discharge was 18% and at 6 months post-discharge was 27%, demonstrating that after discharge these patients are still at high risk of death.

Our study showed that higher comorbidity scores, exposure to NSAIDs, and the need for diuretics were associated with AKI. Diuretics are commonly used in patients with AKI since fluid overload is one of the major complications of decreased kidney function, and diuretics are also used for risk-stratification of AKI progression (furosemide stress test) [33]. Therefore, the association of diuretic use with AKI may be a reflection of more severe forms of AKI (e.g., oliguric/anuric AKI) and not necessarily thought to be contributory to the development or progression of AKI. Few studies have reported clinical parameters associated with AKI in the setting of IE. In multivariable analysis, Ritchie et al., [18] identified independent clinical parameters associated with AKI such as CKD, treatment with nafcillin or oxacillin, treatment with aminoglycoside and vancomycin, and similarly to our study, need of or exposure to loop diuretics. In contrast, we did not find major differences in relation to antibiotic exposure in those with vs. without AKI.

Our study also found that patients with severe AKI (stage ≥2) were more likely to have tricuspid valve involvement and required vasopressor/inotrope support more frequently than patients without AKI. Right-side IE (RSIE) occurs more commonly in persons with substance use disorder, especially persons who inject drugs [34]. In our cohort, patients with tricuspid valve affection (vs. other valves) had more substance use disorder, hepatitis C coinfection and sepsis (74.1% vs. 44.3%; 61.2% vs. 35%; 70.6% vs. 40.4%, respectively, *p* < 0.001 for all), which may have influenced the higher frequency of severe AKI. Lemaire et al., [35] studied 6264 patients with IE who underwent valvular surgery, 809 (12.9%) with a diagnosis of substance use disorder. They found that patients with a substance use disorder were more likely to have post-operative complications, especially infectious complications such as pneumonia (OR 1.4, 95% CI 1.14–1.74), sepsis (OR 1.4, 95% CI 1.16–1.63), and renal complications (OR 1.5, 95% CI 1.23–1.77), defined based on ICD-9-CM diagnosis codes.

We also found that healthcare resource utilization and costs were higher in IE patients with AKI vs. those without AKI. In a different cohort study of 25,495 hospitalized patients who had AKI, Collister et al., reported that AKI was associated with longer length of hospital stay and increased hospital total cost [36]. They also found that incident AKI Stage 1 and 2 resulted in 1.2–1.3 times higher hospital total cost than no AKI while AKI Stage 3 and the need of dialysis were associated with 1.8–2.5 times higher cost. Fleischauer et al., [6] gathered data from 128 hospitals in North Carolina (*n* = 505 patients) and reported healthcare costs related to IE. They found that hospital admissions for drug dependence-associated IE increased twelve-fold from 2010 to 2015 (0.2 to 2.7 cases per 100,000 persons per year). Similarly, the total cost of hospitalization of IE patients increased eighteen-fold in the same period of time (from 1.1 to ~22.2 million). The major limitation of this study is that they only included hospitalizations with drug dependence-related IE listed as an ICD-9/10 CM diagnosis.

Our study has limitations. First, our study is a retrospective, single-center, cohort study of relatively small sample size and therefore the results may not be generalizable to other populations, particularly those with a low prevalence of substance use disorder or more diverse racial backgrounds (>90% of our study population was white). Second, we did not use urine output criteria as part of the AKI definition or examined the clinical etiology of AKI due to lack of data availability. Third, the time point at which antibiotics or other drugs were administered during the course of AKI was not characterized. Therefore, exposure to these drugs may have occurred before, during or after AKI, which can introduce confounding by indication to the interpretation of the results (e.g., more or less exposure in the context of clinically evident AKI rather than a drug-related attributable risk for the AKI event).

Our study has several strengths. First, we examined a large cohort of hospitalized patients with a primary diagnosis of IE compared to other studies examining AKI in the setting of IE [16,18]. Second, we examined distinct clinical parameters (patient-specific, IE-specific and medication exposure) associated with AKI as well as resource utilization and healthcare cost associated with the occurrence of AKI. Our study therefore further contributes to identifying patients with IE at high risk of AKI. Third, our cohort is unique in that is from an area with a high prevalence of opioid use disorder. Finally, our study highlights the need to identify clinical parameters that may inform risk-stratification of AKI and adverse outcomes in this susceptible population.

## 5. Conclusions

Two out of three patients admitted to the hospital with a primary diagnosis of IE had AKI. A higher level of comorbidity was independently associated with AKI. Patients with AKI more frequently received diuretics and patients with severe AKI (stage ≥2) more frequently required pressor/inotrope support. The affection of the tricuspid valve was independently associated with severe AKI (stage ≥2). Patients with severe AKI (stage ≥2) had more frequently major adverse kidney events up to 90 days post-discharge. Further, resource utilization and healthcare cost were significantly higher in patients with AKI vs. those without AKI. Future studies should aim to develop risk-stratification tools for AKI and adverse outcomes in hospitalized patients with IE and therefore guide preventive strategies that ameliorate the burden of complications in this susceptible population.

## Figures and Tables

**Table 1:** Patient characteristics according to acute kidney injury status.

	No AKI	AKI Stage 1	AKI Stage ≥2	*p*
	*n* = 91	*n* = 74	*n* = 104	
**Demographics**				
Age, years, mean ± SD	44.6 ± 15.6	45.5 ± 16.5	45.8 ± 16.8	0.860
Gender, male, n (%)	62 (68.1)	34 (46.0)	64 (61.5)	0.013
Ethnic group, white, *n* (%)	86 (94.5)	71 (96.0)	99 (95.2)	0.939
BMI, kg/m^2^, mean ± SD	27.9 ± 10.9	29.8 ± 11.0	26.9 ± 7.1	0.140
**Comorbidity**				
Elixhauser Score, median (IQ1-IQ3)	4.0 (3.0–6.0)	6.0 (4.0–7.0)	7.0 (5.0–8.0)	<0.001
Diabetes, *n* (%)	23 (25.3)	14 (18.9)	18 (17.3)	0.361
Hypertension, *n* (%)	55 (60.4)	41 (55.4)	69 (66.4)	0.328
Hepatitis B, *n* (%)	1 (1.1)	9 (12.2)	7 (6.7)	0.011
Hepatitis C, *n* (%)	27 (29.7)	38 (51.4)	51 (49.0)	0.006
Congenital heart defect, *n* (%)	6 (6.6)	8 (10.8)	9 (8.7)	0.628
Current substance use disorder, *n* (%)	46 (50.5)	42 (56.8)	55 (52.9)	0.672
Baseline eGFR, mL/min/1.73 m^2^, median (IQ1-IQ3)	105.3 (69.1–127.6)	96.8 (45.5–123.9)	103.2 (71.4–122.9)	0.220
**Medications During Hospitalization**				
Opiods, *n* (%)	3 (3.3)	6 (8.1)	9 (8.7)	0.279
ACEI or ARB, *n* (%)	36 (39.6)	12 (16.2)	21 (20.2)	<0.001
Aminoglycosides, *n* (%)	36 (39.6)	31 (41.9)	52 (50.0)	0.305
Diuretic, *n* (%)	38 (41.8)	51 (68.9)	83 (79.8)	<0.001
NSAIDs, *n* (%)	54 (59.3)	51 (68.9)	77 (74.0)	0.088
Pressor or inotrope, *n* (%)	0 (0.0)	5 (6.8)	19 (18.3)	<0.001
Vancomycin, *n* (%)	76 (83.5)	69 (93.2)	89 (85.6)	0.142
Piperacillin/tazobactam, *n* (%)	30 (33.0)	23 (31.1)	38 (36.5)	0.733
Cefepime, *n* (%)	25 (27.5)	30 (40.5)	39 (37.5)	0.169
Vancomycin + Pip/Tazo, *n* (%)	30 (33.0)	24 (32.4)	36 (34.6)	0.948
Vancomycin + Cefepime, *n* (%)	26 (28.6)	32 (43.2)	37 (35.6)	0.146
**Infective endocarditis characteristics**				
ICU care, *n* (%)	38 (41.8)	39 (52.7)	46 (44.2)	0.346
Sepsis, *n* (%)	32 (35.2)	36 (48.7)	66 (63.5)	<0.001
Osteomyelitis, *n* (%)	14 (15.4)	7 (9.5)	18 (17.3)	0.327
Number of affected cardiac valves,				0.006
0, *n* (%)	45 (49.5)	26 (35.1)	29 (27.9)	
1, *n* (%)	40 (44.0)	42 (56.8)	56 (53.8)	
≥2, *n* (%)	6 (6.5)	6 (8.1)	19 (18.3)	
Affected valve,				
Mitral, *n* (%)	31 (34.1)	27 (37.0)	34 (32.7)	0.837
Aortic, *n* (%)	38 (41.8)	24 (32.9)	35 (33.7)	0.395
Tricuspid, *n* (%)	20 (22.0)	18 (24.7)	47 (45.2)	<0.001
Pulmonic, *n* (%)	1 (1.1)	2 (2.7)	2 (1.9)	0.856
Type of valve, prosthetic, *n* (%)	23 (25.3)	20 (27.0)	33 (31.7)	0.585
Procedures,				
Valve replacement, *n* (%)	16 (17.6)	14 (18.9)	33 (31.7)	0.037
Valve repair, *n* (%)	11 (12.1)	12 (16.2)	16 (15.4)	0.716
Other, *n* (%)	8 (8.8)	3 (4.1)	13 (12.5)	0.165
**Microbiologic data**				
MRSA, *n* (%)	23 (25.3)	20 (27.0)	37 (35.6)	0.286
MSSA, *n* (%)	16 (17.6)	12 (16.2)	27 (26.0)	0.227
MSSE, *n* (%)	0 (0.0)	0 (0.0)	1 (1.0)	0.458
Other *Staphylococcus*, *n* (%)	11 (12.1)	12 (16.2)	8 (7.7)	0.197
*Streptococcus*, *n* (%)	24 (26.4)	13 (17.6)	17 (16.3)	0.155
*Enterococcus*, *n* (%)	12 (13.2)	15 (20.3)	16 (15.4)	0.460
Gram-negative rods, *n* (%)	10 (11.0)	13 (17.6)	15 (14.4)	0.492
*Rothia*, *n* (%)	2 (2.2)	2 (2.7)	1 (1.0)	0.659
*Candida sp*., *n* (%)	1 (1.1)	5 (6.8)	9 (8.7)	0.068
Negative culture, *n* (%)	4 (4.4)	4 (5.4)	2 (1.9)	0.428

Abbreviations: ACEI (angiotensin-converting-enzyme inhibitor), AKI (acute kidney injury), ARB (angiotensin receptor blocker), BMI (body mass index), eGFR (estimated glomerular filtration rate), ICU (intensive care unit), IQ (interquartile), MRSA (methicillin-resistant *Staphylococcus aureus*), MSSA (methicillin-susceptible *Staphylococcus aureus*), MSSE (methicillin-susceptible *Staphylococcus epidermidis*), NSAID (nonsteroidal anti-inflammatory drug), Pip/Tazo (piperacillin/tazobactam), SD (standard deviation).

**Table 2:** Multivariable logistic regression models of AKI stage ≥1 and AKI stage ≥2 as the dependent variables and relevant clinical parameters as the independent variables.

	AKI Stage ≥1 vs. No AKI		AKI Stage ≥2 vs. AKI Stage 1 or No AKI	
	OR	95% CI	OR	95% CI
ACEI or ARB, Yes vs. No	0.28 **	0.13–0.57	0.66	0.33–1.32
Tricuspid valve affected, Yes vs. No	1.71	0.84–3.46	2.97 **	1.59–5.51
Use of diuretic, Yes Vs. No	3.18 **	1.63–6.23	2.04 *	1.04–4.01
Hepatitis B coinfection, Yes vs. No	6.57	0.78–55.16	0.61	0.19–1.97
Use of NSAID, Yes vs. No	1.95 *	1.01–3.76	1.72	0.90–3.25
Pressor or Inotrope need, Yes vs. No	-	-	4.94 *	1.52–16.01
Diabetes, Yes vs. No	0.47	0.21–1.06	0.55	0.26–1.19
Elixhauser score, per 1-unit score	1.35 **	1.16–1.56	1.35 **	1.17–1.55

Candidate variables for the multivariable models included age, baseline eGFR, hepatitis B or C coinfection, use of diuretic, use of ACEI or ARB, affection of the tricuspid valve, use of NSAID, need of pressor or inotrope, Elixhauser comorbidity score, history of diabetes, and zip code with the highest percentage of population below poverty level. C-statistic: 0.830, 0.788 for AKI stage ≥1 vs. no AKI and AKI stage ≥2 vs. AKI stage 1 or no AKI, respectively. * *p* < 0.05; ** *p* < 0.01. Abbreviations: ACEI (angiotensin-converting enzyme inhibitor), ARB (angiotensin-receptor blocker), NSAID (nonstereoidal anti-inflammatory drug).

**Table 3:** Study outcomes in hospitalized patients with infective endocarditis according to acute kidney injury status.

	No AKI *n* = 91	AKI Stage 1 *n* = 74	AKI Stage ≥2 *n* = 104	*p* Value
**Clinical outcomes**				
Hospital mortality, *n* (%)	14 (15.4)	8 (10.8)	20 (19.2)	0.312
Readmission due to IE, (%)	4 (4.4)	4 (5.4)	3 (2.9)	0.687
MAKE50 *, *n* (%)	35 (38.5)	40 (54.1)	62 (59.6)	0.011
MAKE25 ^#^, *n* (%)	55 (60.4)	51 (68.9)	77 (74.0)	0.125
**Healthcare cost outcomes**				
Total direct hospitalization cost, dollars, median (IQ1-IQ3)	17,069 (6722–31,910)	37,111 (20,100–58,258)	61,357 (34,164–88,495)	<0.001
Hospital length of stay, days, median (IQ1-IQ3)	9.0 (5.0–17.5)	23.0 (12.0–43.8)	34.5 (16.8–48.0)	<0.001
Telemetry bed days, median (IQ1-IQ3)	3.0 (0.0–10.0)	8.0 (2.0–17.0)	9.5 (1.0–20.0)	<0.001
ICU length of stay, median (IQ1-IQ3)	0.0 (0.0–2.0)	1.5 (0.0–5.0)	6.0 (1.5–14.5)	<0.001

* MAKE50 = Major Adverse Kidney Event, which includes mortality at or within 90 days of hospital discharge, continued RRT-dependence at 90 days after hospital discharge, or recovery of at least 50% of baseline eGFR within 90 days of discharge.^#^ MAKE25 = Major Adverse Kidney Event, which includes mortality at or within 90 days of hospital discharge, continued RRT-dependence at 90 days after hospital discharge, or recovery of at least 25% of baseline eGFR within 90 days of discharge. Abbreviations: AKI (acute kidney injury), ICU (intensive care unit), IQ (interquartile range), MAKE (major adverse kidney event).

**Table 4:** Multivariable logistic regression models of major adverse kidney events as the dependent variable and relevant clinical parameters as the independent variables.

	MAKE50		MAKE25	
	OR	95% CI	OR	95% CI
Sepsis, Yes vs. No	2.03 **	1.23–3.36	1.56	0.91–2.66
AKI Severity				
Stage ≥2 vs. No AKI	1.97	1.09–3.58	1.66	0.89–3.10
Stage 1 vs. No AKI	1.74	0.92–3.28	1.37	0.71–2.64

Candidate variables for the multivariable models included age, gender, history of hypertension, sepsis during indexed admission, affection of tricuspid valve, use of NSAID, use of ACEI or ARB, and AKI severity. C-statistic: 0.641, 0.590 for MAKE50 and MAKE25 respectively. ** *p* < 0.01. MAKE50 = Major Adverse Kidney Event, which includes mortality at or within 90 days of hospital discharge, continued RRT-dependence at 90 days after hospital discharge, or no recovery of at least 50% of baseline eGFR within 90 days of discharge. MAKE25 = Major Adverse Kidney Event, which includes mortality at or within 90 days of hospital discharge, continued RRT-dependence at 90 days after hospital discharge, or no recovery of at least 25% of baseline eGFR within 90 days of discharge. Abbreviations: AKI (acute kidney injury), KDIGO (kidney disease improving global outcomes), MAKE (major adverse kidney event).
